# Respiratory variation in carotid peak systolic velocity predicts volume responsiveness in mechanically ventilated patients with septic shock: a prospective cohort study

**DOI:** 10.1186/s13089-015-0029-1

**Published:** 2015-06-26

**Authors:** Miguel Á Ibarra-Estrada, José A López-Pulgarín, Julio C Mijangos-Méndez, José L Díaz-Gómez, Guadalupe Aguirre-Avalos

**Affiliations:** Intensive Care Unit, Hospital Civil de Guadalajara “Fray Antonio Alcalde”, Hospital 278, El Retiro, Specialties Building, Floor 1, Guadalajara, Jalisco 44280 Mexico; Centro Universitario de Ciencias de la Salud, Universidad de Guadalajara, Independencia Oriente, 44340 Guadalajara, Jalisco Mexico; Departments of Critical Care Medicine, Anesthesiology, and Neurosurgery, Mayo Clinic, 4500 San Pablo Road, Jacksonville, FL USA

**Keywords:** Sepsis, Carotid Doppler peak velocity, Pulse pressure variation, Passive leg raising, Transpulmonary thermodilution

## Abstract

**Background:**

The evaluation of fluid responsiveness in patients with hemodynamic instability remains to be challenging. This investigation aimed to determine whether respiratory variation in carotid Doppler peak velocity (ΔCDPV) predicts fluid responsiveness in patients with septic shock and lung protective mechanical ventilation with a tidal volume of 6 ml/kg.

**Methods:**

We performed a prospective cohort study at an intensive care unit, studying the effect of 59 fluid challenges on 19 mechanically ventilated patients with septic shock. Pre-fluid challenge ΔCDPV and other static or dynamic measurements were obtained. Fluid challenge responders were defined as patients whose stroke volume index increased more than 15 % on transpulmonary thermodilution. The area under the receiver operating characteristic curve (AUROC) was compared for each predictive parameter.

**Results:**

Fluid responsiveness rate was 51 %. The ΔCDPV had an AUROC of 0.88 (95 % confidence interval (CI) 0.77–0.95); followed by stroke volume variation (0.72, 95 % CI 0.63–0.88), passive leg raising (0.69, 95 % CI 0.56–0.80), and pulse pressure variation (0.63, 95 % CI 0.49–0.75). The ΔCDPV was a statistically significant superior predictor when compared with the other parameters. Sensitivity, specificity, and positive and negative predictive values were also the highest for ΔCDPV, with an optimal cutoff at 14 %. There was good correlation between ΔCDPV and SVI increment after the fluid challenge (*r* = 0.84; *p* < 0.001).

**Conclusions:**

ΔCDPV can be more accurate than other methods for assessing fluid responsiveness in patients with septic shock receiving lung protective mechanical ventilation. ΔCDPV also has a high correlation with SVI increase after fluid challenge.

## Background

In a patient with acute hemodynamic instability, a fluid challenge will cause an increase in stroke volume, according to the Frank-Starling curve [1]. This increase in stroke volume has a salutary effect because it improves tissue perfusion. In contrast, higher hydrostatic pressures in the vascular system predispose the patient to edema, organic dysfunction, and increased risk of in-hospital mortality [2, 3]. Relative hypovolemia has been described in the setting of septic shock. However, only 50 % of patients with hemodynamic instability are fluid responsive [4, 5]. Therefore, expeditious fluid resuscitation is advised, and clinicians must always weigh the benefits and risks of intravenous fluids [2, 6].

Currently, both static and dynamic parameters are utilized for prediction of fluid responsiveness. Static parameters (e.g., central venous pressure and pulmonary artery occlusion pressure) are much less reliable than dynamic parameters, which are based on respirophasic variation in stroke volume (e.g., pulse pressure variation and changes in aortic blood flow) [7]. Most common dynamic parameters are invasive (arterial and/or central venous cannulation is required) and expensive. Echocardiography is a well-established method for evaluating fluid responsiveness [5, 8, 9]. Nevertheless, measurement of left ventricular outflow tract velocities for the estimation of stroke volume is labor intense, requires specific training for adequate performance, and is not easily reproducible or obtainable [8, 10]. Thus, alternative methods, including brachial or carotid artery velocity, have been examined as surrogates for stroke volume in the non-septic shock patient population [11, 12]. Moreover, most predictive indices for volume responsiveness are not validated in patients receiving lung protective ventilatory strategies. The aim of this study was to determine if respiratory variation in carotid Doppler peak velocity (ΔCDPV) can predict fluid responsiveness in patients with septic shock and lung protective mechanical ventilation.

## Methods

### Patients

This was a single-center, prospective, cohort study. Inclusion criteria were mechanical ventilation, septic shock, and hemodynamic instability for which the attending intensivist determined the need for fluid challenge based on signs of inadequate tissue perfusion according to Surviving Sepsis Campaign recommendations [13]. The investigation was conducted in a medical/surgical intensive care unit and tertiary academic hospital from May 2014 through October 2014. Exclusion criteria were age under 18 years, non-septic origin of shock, known heart failure, valvular disease or arrhythmia, intra-abdominal hypertension, peripheral arterial disease, common carotid artery stenosis greater than 50 % (systolic peak velocity >182 cm/s and/or diastolic velocity >30 cm/s by Doppler ultrasound), spontaneous respiratory efforts, and utilization of colloids other than albumin for the fluid challenge [14]. Volume controlled mechanical ventilation was performed with tidal volumes at 6 ml/kg of predicted body weight. We usually administer fluid challenges with normal saline at a 7 mL/kg dose over a 30-min period and perform thermodilution before and after each challenge. The Institutional Review Board at Hospital Civil de Guadalajara deemed the investigation to be of minimal risk and waived the need for written consent.

### Measurements and volume responsiveness

**Fig. 1:**
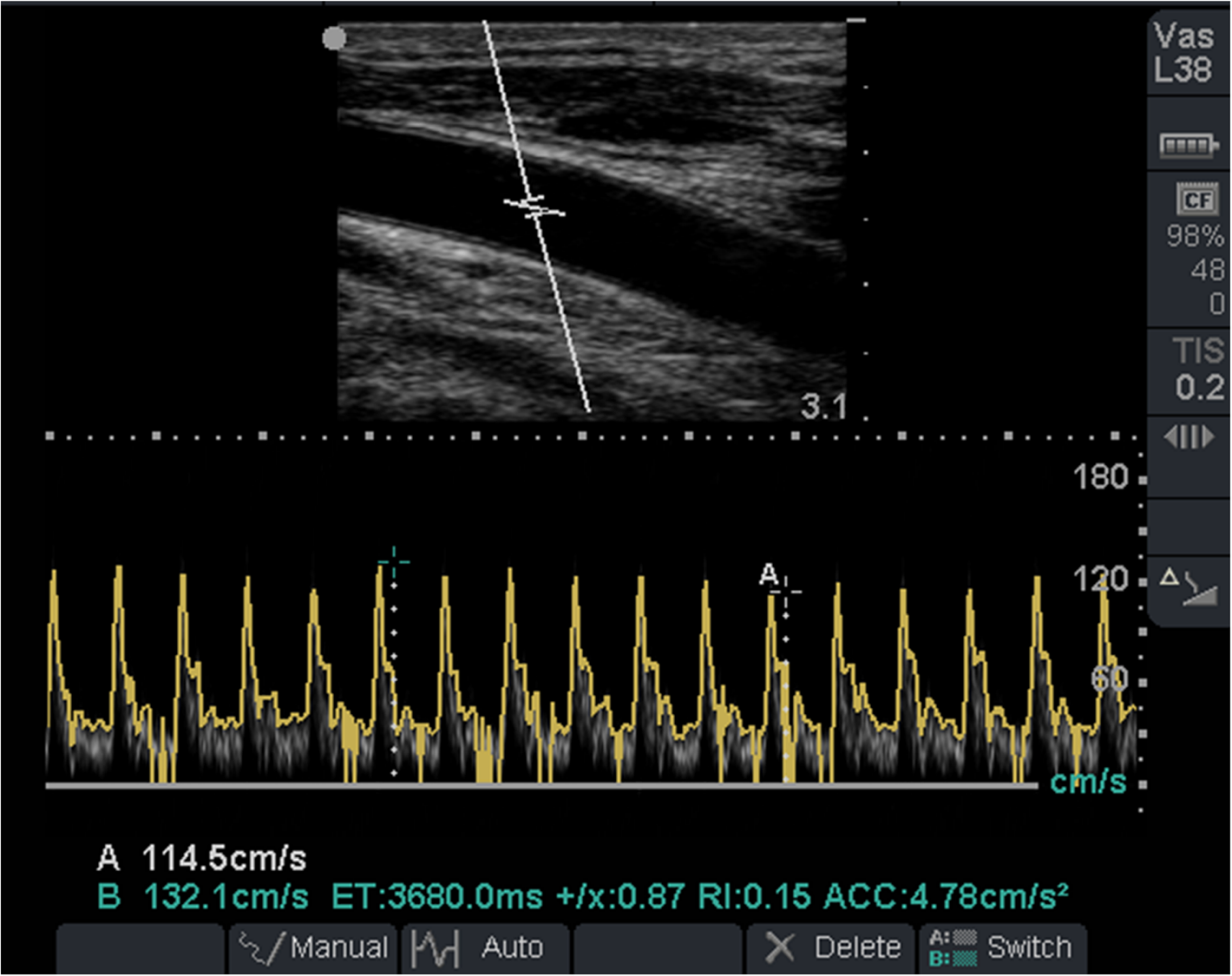
Measurement of variation in carotid peak systolic velocity. At 14 % in this patient

Before each fluid challenge, carotid peak systolic velocity was measured with a Micromaxx System (Sonosite, WA, USA), using a 5–10-mHz linear array transducer. After procuring a longitudinal view of the common carotid artery, pulsed Doppler analysis at 2 cm from the bifurcation was performed. The sample volume was positioned at the center of the vessel, with angulation at no more than 60°. Maximum and minimum peak systolic velocities were obtained in a single respiratory cycle (Fig. 1), and the ΔCDPV was calculated with the following formula: (MaxCDPV − MinCDPV) / [(MaxCDPV + MinCDPV) / 2] × 100, expressed as a percentage [12]. Two investigators with previous formal training in critical care ultrasound estimated the ΔCDPV. These investigators were blinded to each other’s results and to all other variables. The mean of both measurements obtained by the two investigators was used. In addition, the same investigators evaluated the adequate procurement of transthoracic echocardiographic windows for estimation of the stroke volume. Pulse pressure variation (PPV) was calculated with the formula: PPV (%) = 100 × (Pp max − Pp min) / [(Pp max + Pp min) / 2] [15], with pressures measured from a femoral arterial catheter with the v2.6e monitor (Phillips Healthcare, Eindhoven, the Netherlands). The passive leg raising (PLR) test was performed as previously reported [16] before each challenge by placing the patient’s head and upper torso upright at 45°. This was followed by a flat supine position and raising both legs to a 45° angle from the bed, while measuring the SVI before and after the maneuver. The highest SVI from the first 3 min after the test was taken, and the percentage increase in SVI with the PLR was recorded. Inferior vena cava diameter (IVC-d) measurement was performed with a two-dimensional view at a sub-xyphoidal long axis, approximately 2 cm caudal to the hepatic vein inlet. Maximum and minimum diameters over a single respiratory cycle were recorded, and respiratory variation in inferior vena cava diameter (ΔD-IVC) was calculated with the formula: (Max IVC-d − Min IVC-d) / [(Max IVC-d + Min IVC-d) / 2]. Transpulmonary thermodilution was performed before and after each fluid challenge with the Pulse Contour Cardiac Output system (Pulsion Medical Systems, Münich, Germany) to obtain an automated SVI, stroke volume variation (SVV), and other variables. Patients with an increase of more than 15 % in the SVI after the fluid challenge were classified as “responders”, and those with an increase of less than 15 % in the SVI or those with no increase were classified as “non-responders.”

### Statistical analysis

Continuous variables were reported as the mean (standard deviation) if they were normally distributed or the median (interquartile range) if they were not normally distributed, using the Shapiro–Wilk test. Preload indices were compared in responders and non-responders using the Mann–Whitney test. Categorical variables were expressed as the number of measurements (%) and were compared by the chi-squared test. For analyzing the trend in response at repeated fluid challenges per patient, we used the Cochran–Armitage test. We constructed receiver operating characteristic (ROC) curves for static and dynamic indices of preload to determine the ability to predict fluid responsiveness, and their area under the curve was compared using the Hanley–McNeil test [17]. Optimal cutoff values were obtained with the greatest sum of sensitivity and specificity using the Youden index [18]. The relationship between preload indices and changes in SVI after the fluid challenge was estimated with Spearman’s correlation coefficient test. We determined inter-observer reproducibility for ΔCDPV by using the Bland–Altman Plot, described as mean bias [19]. Inter-rater agreement was calculated with the kappa statistic and a 95 % confidence interval (CI) [20]. Assuming a fluid responsiveness rate of 50 %, we determined that 36 measurements would be needed to detect differences of 0.30 between the area under the receiver operating characteristic (AUROC) curve of central venous pressure (0.55) [4] and ΔCDPV (0.85) [11], with an 80 % power and type I error of 5 %. For all tests, *p* values were two-sided, and a *p* value lower than 0.05 was considered statistically significant. We used MedCalc (Ver 13.2, Mariakerke, Belgium) for calculating the sample size and for the statistical analysis.

## Results

A total of 59 fluid challenges were performed in 19 patients, with a responsiveness rate of 51 %. In eight patients (40 %), the velocity-time integral at the left ventricle outflow tract was not obtained due to an unfavorable transthoracic echocardiographic window. Baseline characteristics of the patients are shown in Table 1.

**Table 1:** Characteristics and baseline parameters before all fluid challenges, between responders and non-responders

Variable	Responders	Non-responders	*P* value
	*n* = 30	*n* = 29	
Age (years)	51 (38–58)	53 (38–57)	0.65
Gender (male, %)	20 (67)	17 (59)	0.71
BSA (m^2^)	1.65 (1.50–1.80)	1.60 (1.57–1.80)	0.72
ARDS (%)	22 (73)	21 (72)	0.83
AKI (%)	15 (50)	14 (48)	0.88
Shock diagnosis (hours)	17.5 (13–23)	27 (21.7–35)	0.0001
MAP (mmHg)	61 (60–62.8)	63 (61–64.2)	0.053
HR (beats/min)	119 (117–123)	121 (115–124)	0.63
UO (ml/kg/h)	0.4 (0.2–0.6)	0.4 (0.2 – 0.8)	0.34
Arterial Lactate (mmol/L)^a^	5.3 ± 1.6	4.7 ± 1.7	0.20
ScvO2 (%)	64 ± 10.7	67 ± 10.2	0.26
SVI (ml/m^2^)	16 (14–18)	17 (15–19)	0.09
NE dose (mcg/kg/min)	0.33 (0.21–0.57)	0.40 (0.33–0.54)	0.36
Fluid balance (L)^a^	2.12 ± 0.70	2.62 ± 0.75	0.01
Fluid loads (n)	2 (2–3)	2 (2–3)	0.19
SOFA (score)	10 (8–13)	15 (13–18)	<0.0001
PEEP (cm/H_2_O)	6 (5–7)	6 (5–7)	0.71
Plateau pressure (cm/H_2_O)^a^	24 ± 3	24 ± 3	0.98
Tidal volume (ml/kg)	6 (6.0–6.3)	6 (6.0–6.3)	0.96

All data are expressed as median (interquartile range), except those marked with ^a^, which are expressed as mean (standard deviation)

*BSA* body surface area, *ARDS* acute respiratory distress syndrome, *AKI* acute kidney injury, *MAP* mean arterial pressure, *HR* heart rate, *UO* urinary output, *ScvO2* oxygen saturation at central venous blood, *SVI* stroke volume index, *NE* norepinephrine, *SOFA* Sequential Organ Failure Assessment score, range from 0 to 24, with higher scores indicating a greater risk of mortality, *PEEP* positive end-expiratory pressure

### Predictors of fluid responsiveness

**Fig. 2:**
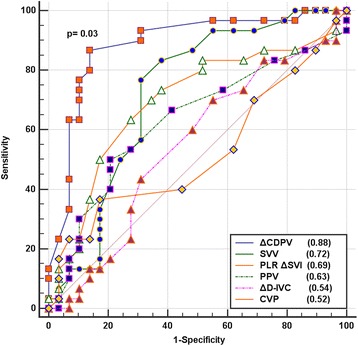
Areas under the receiver operating characteristic curve of predictors of fluid responsiveness. The *p* value indicates comparison between respiratory variation in carotid peak velocity and stroke volume variation (SVV) with the Hanley–McNeil test

The ΔCDPV, SVV, SVI increment following the PLR test, and PPV were significantly higher in responders than in non-responders. There was no significant difference in the ΔD-IVC or in any of the static parameters (Table 2). Among dynamic variables, ΔCDPV had the highest AUROC (0.88, *p* < 0.001; 95 % CI 0.77–0.95) (Table 3), with an optimum cutoff value of greater than 14 % based on the Youden index. Using the Hanley–McNeil test, ΔCDPV was significantly superior to the other variables, (*p* = 0.03 versus SVV, *p* = 0.01 versus PLR, *p* = 0.001 versus PPV, and *p* < 0.001 versus ΔD-IVC; Fig. 2). ΔCDPV showed the highest sensitivity and specificity, as well as positive and negative predictive values (Table 4).

**Table 2:** Baseline differences in predictors of fluid responsiveness between responders and non-responders

Variable	Responders	Non-responders	*P* value
	*n* = 30	*n* = 29	
ΔCDPV (%)	21 (17–23)	13 (10–14)	<0.0001
SVV (%)	18.5 (17–22)	14 (11–18)	0.003
PLR Delta SVI (%)	16.5 (13–18)	13 (10.5–16)	0.01
PPV (%)	14.5 (11–19)	12 (10–14)	0.08
ΔD-IVC (%)	15 (12–17)	14 (11–18)	0.54
GEDI (ml/m^2^)^a^	472 ± 115	490 ± 102	0.53
IVC-d (mm)^a^	10.9 ± 3.8	10.5 ± 3.7	0.69
CVP (mmHg)	8 (6–10)	8 (6–9)	0.78

Data are expressed as median (interquartile range), except those marked with ^a^, which are expressed as a mean (standard deviation)

*ΔCDPV* respiratory variation in carotid Doppler peak velocity, *SVV* stroke volume variation, *PLR Delta SVI* rise in stroke volume index after passive leg elevation test, *PPV* pulse pressure variability, *ScvO2* oxygen saturation at central venous blood, Δ*D-IVC* respiratory variation in inferior vena cava diameter, *GEDI* global end-diastolic index, *IVC-d* inferior vena cava diameter, *CVP* central venous pressure

**Table 3:** Correlations between predictors and the fluid challenge-induced change in the stroke volume index

Variable	Correlation coefficient (95 % CI)	*p* value	AUROC (95 % CI)	*p* value
ΔCDPV (%)	0.84 (0.74–0.90)	<0.001	0.88 (0.77–0.95)	<0.001
SVV (%)	0.24 (−0.009–0.47)	0.05	0.72 (0.59–0.83)	0.001
PLR Delta SVI (%)	0.24 (−0.01–0.46)	0.06	0.69 (0.56–0.80)	0.005
PPV (%)	0.02 (−0.23–0.27)	0.08	0.63 (0.49–0.75)	0.007
ΔD-IVC (%)	−0.02 (−0.27–0.23)	0.84	0.54 (0.41–0.67)	0.50
GEDI (ml/m^2^)	0.02 (−0.23–0.27)	0.85	0.55 (0.41–0.68)	0.48
IVC-d (mm)	0.04 (−0.21–0.29)	0.74	0.52 (0.39–0.65)	0.75
CVP (mmHg)	−0.09 (−0.34–0.16)	0.48	0.52 (0.38–0.65)	0.78

*AUROC* areas under the receiver operating characteristic curves, Δ*CDPV* respiratory variation in carotid Doppler peak velocity, *SVV* stroke volume variation, *PLR Delta SVI* rise in stroke volume index after passive leg elevation test, *PPV* pulse pressure variation, *ScvO2* oxygen saturation at central venous blood, Δ*D-IVC* respiratory variation in inferior vena cava diameter, *GEDI* global end-diastolic index, *IVC-d* inferior vena cava diameter, *CVP* central venous pressure

**Table 4:** Cutoffs and diagnostic performances of significant predictors

Variable	Cutoff	Sensitivity	Specificity	PPV^a^	NPV
		(95 % CI)	(95 % CI)	(95 % CI)	(95 % CI)
ΔCDPV (%)	>14	86 (69–96)	86 (68–96)	86 (69–96)	85 (67–95)
SVV (%)	>16	76 (57–90)	68 (49–84)	71 (52–85)	73 (52–88)
PLR Delta SVI (%)	>15	63 (44–80)	72 (52–87)	70 (49–86)	65 (46–81)
PPV (%)	>14	50 (31–68)	79 (60–92)	71 (48–88)	60 (43–75)

Only the indices with an AUROC >0.6 and *p* < 0.05 were included. Cutoffs estimated by Youden index

*NPV* negative predictive value, Δ*CDPV* carotid peak velocity variation, *SVV* stroke volume variation, *PLR Delta SVI* rise in stroke volume index after passive leg elevation test, *PPV* pulse pressure variation

^a^Positive predictive value

Because it may be arguable as to what cutoff points of increase in SVI is truly clinically meaningful, we calculated ROC curves of the main indices taking an increase in SVI >10 %, instead of >15 % as cutoff. The results were similar, as ΔCDPV maintained the greatest AUROC (0.90, *p* = <0.001).

Responders had a significantly higher median rise in the SVI after the fluid challenge compared to non-responders (42 versus 9.3 %, *p* < 0.001), notwithstanding pre-challenge SVI was not different (16 ml/m^2^ versus 17 ml/m^2^, *p* = 0.09). As seen in Table 5, at repeated measures analysis, there was no significant trend in the progressive number of fluid challenges per patient and responsiveness rate (*p* = 0.29) or ΔCDPV (*p* = 0.32). Median in time between fluid challenges per patient was 4 h (IQR 3.2–5). The presence of acute respiratory distress syndrome or acute kidney injury was not associated with a lack of response to fluid challenge. There was no newly detected carotid stenosis or diminished ejection fraction. The mean time to obtain ΔCDPV was 54 s (SD, 3.9 s).

**Table 5:** Repeated measures analysis

	Fluid challenge (#)					*p* value for trend^a^
	#1	#2	#3	#4	#5	
Response (*n*)	11	9	7	2	1	0.29
No-response (*n*)	8	8	8	3	2	
ΔCDPV % (SD)	19.4 (6.7)	16.4 (7.4)	14.8 (6.3)	14.5 (6.1)	16.3 (4.9)	0.32

^a^Cochran–Armitage test for response/no-response. Repeated measures ANOVA for ΔCDPV

### Prediction of the hemodynamic effects of fluid challenge

**Fig. 3:**
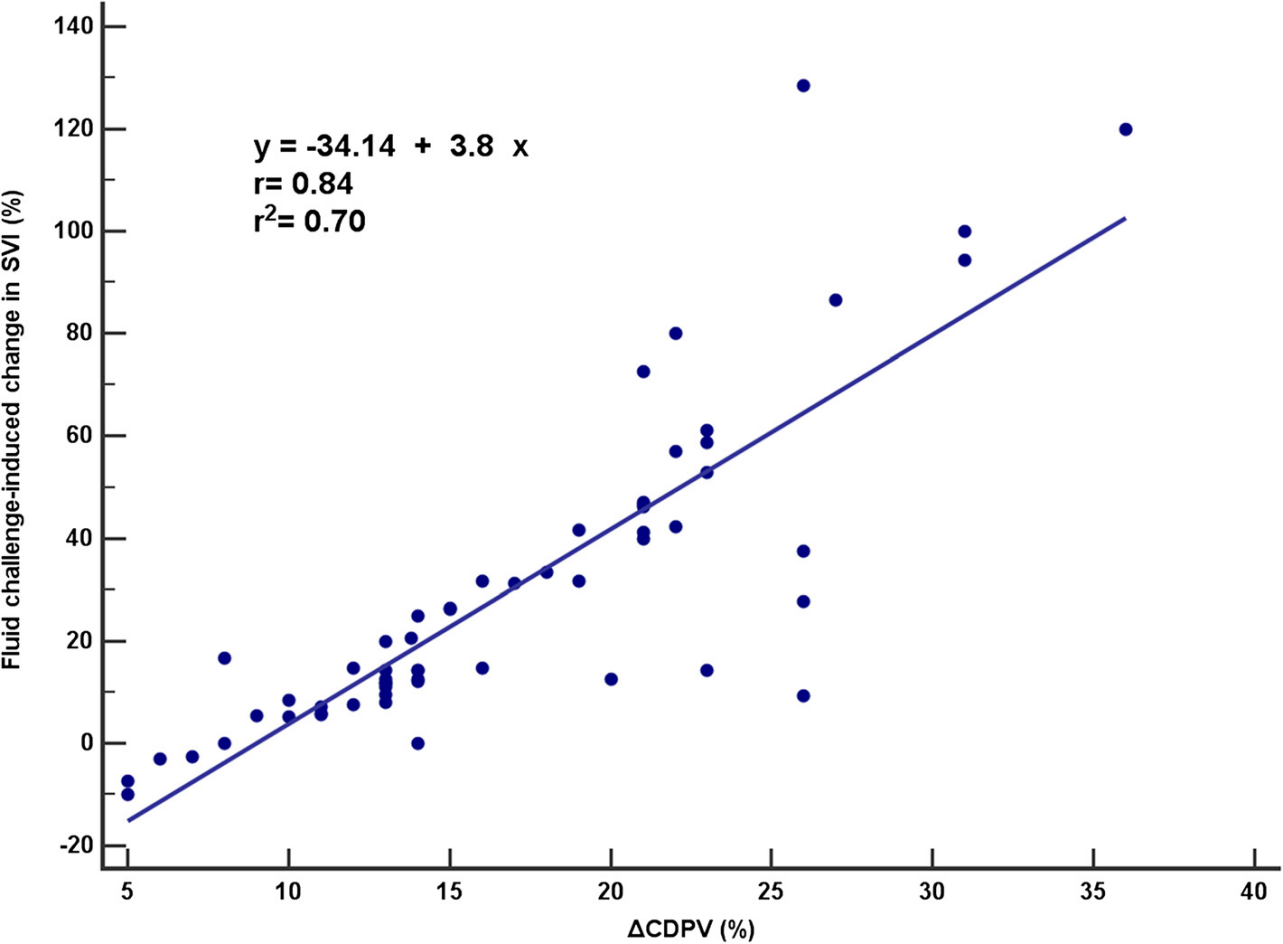
Correlation between variation in respiratory carotid peak systolic velocity and fluid challenge-induced changes in the stroke volume index

Only ΔCDPV was positively correlated with a fluid challenge-induced change in the SVI, and ΔCDPV had the highest correlation coefficient (*r* = 0.84, *p* < 0.001, 95 % CI 0.74–0.90). A regression formula for predicting a rise in the SVI after fluid challenge was obtained (Fig. 3). The correlation between SVV and SVI increase due to fluid challenge was low (*r* = 0.24, *p* = 0.058, 95 % CI −0.009–0.47, *r*^2^ = 0.06). There was no significant correlation between the other indices and change in the SVI.

### Reproducibility and agreement of ΔCDPV

**Fig. 4:**
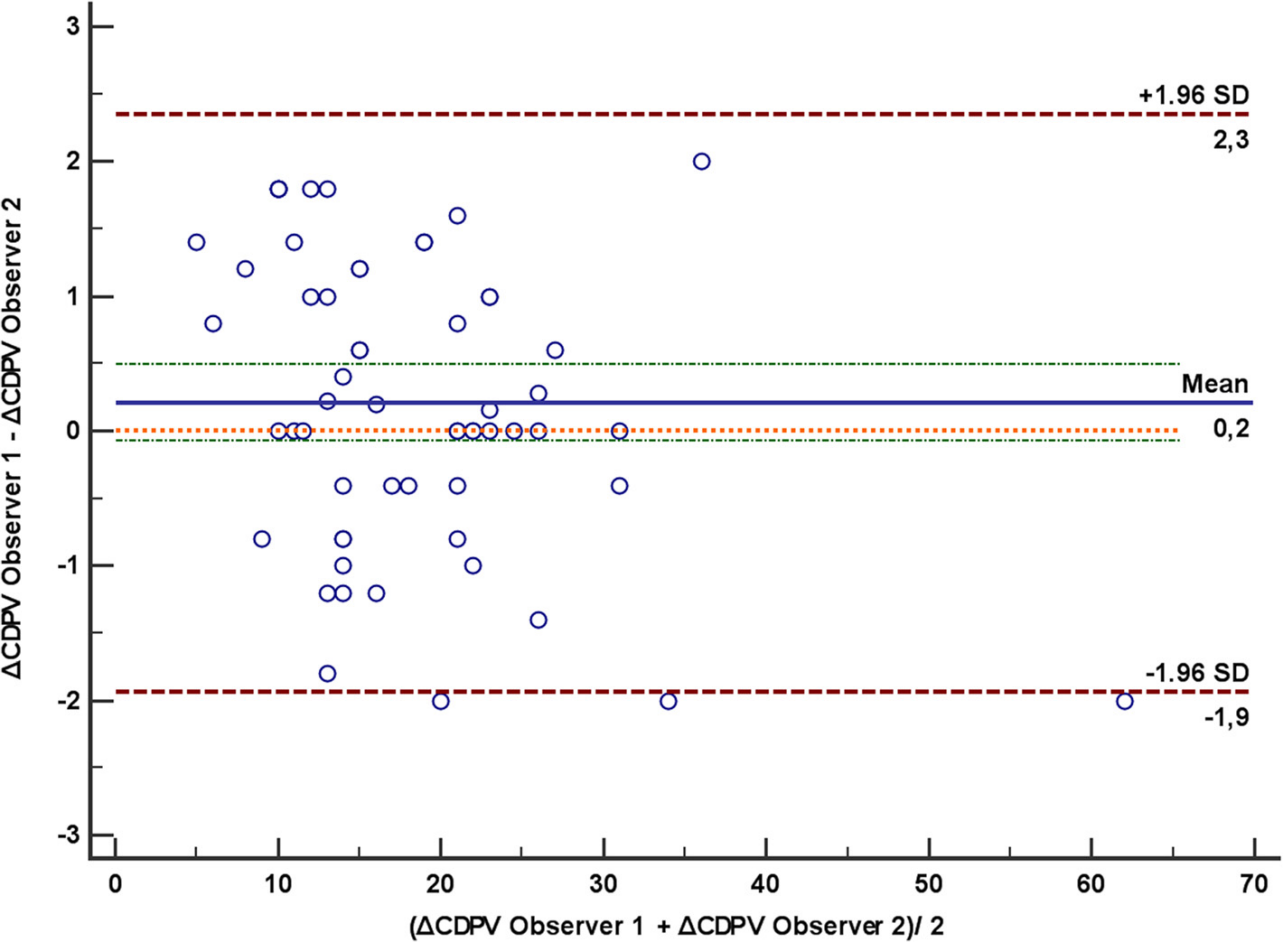
Bland–Altman plot for measurements of both observers. There was a mean bias of 0.2, with limits of agreement between −1.9 and 2.3

Bland–Altman analysis showed good concordance between estimation of ΔCDPV by the two investigators, with a mean bias of 0.2 and limits of agreement between −1.9 and 2.3 (Fig. 4). The inter-observer variability was good, with a kappa statistic of 0.87 (95 % CI 0.84–0.91).

## Discussion

The principal finding of this study is that ΔCDPV is easily obtainable and more accurate than conventional methods (central venous pressure, respiratory variation in inferior vena cava diameter, pulse pressure variation) for assessing fluid responsiveness in mechanically ventilated patients with septic shock. Furthermore, the ΔCDPV has a high correlation with SVI increase after fluid challenge. To our knowledge, this is the first investigation that utilizes the ΔCDPV as a predictor of fluid responsiveness in patients with lung protective mechanical ventilation and septic shock.

In our study, up to 40 % of the patient population had technically difficult echocardiographic apical views, which limited the measurement of the velocity time integral at the left ventricle outflow tract. Hence, alternative non-invasive and practical methods for assessment of fluid responsiveness in septic shock should be investigated. This is a limitation of point-of-care echocardiography in the ICU because the procurement of different acoustic windows varies and presents different degrees of difficulty for mastery [21]. Moreover, Young et al. demonstrated that TTE failed to evaluate the ejection fraction in 69 % of the patients in the ICU [22]. Furthermore, measurement of carotid peak flow can be rapidly performed with less difficulty than for other echocardiographic variables [16].

Other authors have explored the applicability of echocardiography in mechanically ventilated patients. Feissel et al. [23] reported high accuracy, sensitivity (100 %), and specificity (89 %) of respiratory variation in aortic blood velocity (cutoff value higher than 12 %) for prediction of fluid responsiveness in septic patients receiving mechanical ventilation. Similarly, Monnet et al. [24] showed the respiratory variation in aortic peak velocity (cutoff value higher than 13 %) as a predictor of fluid responsiveness with an AUROC of 0.82 and sensitivity and specificity of 80 and 72 %, respectively. However, these studies utilized an invasive method such as transesophageal echocardiography, and patients with an inadequate aortic blood flow signal were excluded. Moreover, not all patients had septic shock [24], and they were ventilated with tidal volumes greater than 6 mL/kg [23, 24].

Monge Garcia et al. [12] demonstrated that the variation in brachial artery peak velocity was a good predictor of fluid responsiveness, with a sensitivity and specificity of 74 and 95 %, respectively. The AUROC was 0.88, similar to our method. In contrast to our study, only half of the patients in their study were septic, and they used the Flo Trac/Vigileo system. This system is a non-calibrated monitoring device for which the accuracy for tracking changes in the cardiac index has come under question. A recent study compared the arterial pressure waveform-derived cardiac index provided by the Vigileo system and pulse contour-derived cardiac index provided by the PiCCO device. The former device performed poorly, with lack of response to therapeutic interventions (volume expansion and vasopressor administration). We used the PiCCO system in this investigation [25].

The preferential diversion of blood flow toward the carotid arteries, away from the peripheral arteries, is a relevant pathophysiological consideration in patients suffering from shock [26]. Considering these facts as well as the flaws in radial artery-based monitoring [27, 28], Song et al. [11] evaluated peak velocity variation at the carotid artery. They showed an AUROC of 0.85, with a threshold value for fluid responsiveness of 11 % (sensitivity and specificity of 0.83 and 0.82). These results are similar to our study. However, their study population primarily consisted of coronary artery disease patients. In comparison to Song’s study, we showed a higher correlation between ΔCDPV and a fluid challenge-induced SVI increase (*r* = 0.84 versus *r* = 0.63) [11]. This finding could be explained by the higher mean age in their study. Perhaps their patients have lower vessel compliance and/or reduced cardiac reserve with concomitant coronary artery disease.

Recently, Marik et al. [26] evaluated the blood flow changes in the carotid artery after a PLR maneuver as a predictor of fluid responsiveness in 34 hemodynamically unstable patients. Among these patients, 65 % presented with severe sepsis/septic shock, and 56 % required mechanical ventilation. The increase in carotid blood flow of greater than or equal to 20 % after PLR was found to have a sensitivity and specificity of 94 and 86 %, respectively. The AUROC curve was, however, not estimated. Their method differed from our study because they not only measured the systolic peak velocity but also calculated the variation in blood flow, which is more labor intensive because it requires measurement of the vessel diameter. In addition, they coupled Doppler estimations with a PLR maneuver. In contrast, we obtained an adequate discriminatory performance with a simpler method, using only the peak systolic velocities at a single respiratory cycle, which showed good inter-observer agreement and reproducibility.

There has been a recent interest in the response to fluid administration over time in patients with shock. Nunes et al. [29] highlighted the limited success of volume resuscitation in patients with circulatory shock after initial resuscitation (>6 h). Thus, a fluid challenge response is not always sustained. We found similar results, as the time from diagnosis of septic shock had a median of 22 h. Another relevant finding in the aforementioned study is that after a fluid challenge, the cardiac index with crystalloid (500 mL infused over a 30-min period) decreased toward baseline values 60 min after infusion, even in responders. Hence, we performed our analysis with the number of “measurements” rather than the number of “patients.” Most patients, who are classified as responders, could have received additional fluid challenges at any time for different clinical contexts (e.g., fluid balance and challenges, presence of organic failure, and different vasopressor dosages).

The performance of PPV and SVV for prediction of fluid responsiveness has been previously reported, with both sensitivity and specificity higher than 90 % [15, 30]. However, in our study, its predictive accuracy was lower than expected. A possible explanation for this difference could be the aforementioned preferential diversion of blood flow toward the carotid arteries and away from the peripheral arteries [26], as well as that the fluid challenges in the study were administrated to mechanically ventilated patients with tidal volumes greater than or equal to 8 mL/kg [15], whereas this variable is not specified in the other studies [31, 32]. We followed a lung protective ventilator strategy with tidal volumes of 6 mL/kg in all patients [33], and high tidal volume influences the hemodynamic effects of a fluid challenge [34].

### Limitations

This study has some limitations. As a respirophasic dynamic index, ΔCDPV does not apply to patients with spontaneous breathing, arrhythmias, valvular disorders, significant heart failure, and common carotid stenosis. Nonetheless, measurement of ΔCDPV is a reliable method, without the inherent risk of central artery cannulation, which is present for thermodilution or pulse contour analysis systems. Additionally, there is no need to raise the patient’s legs, which is a time-consuming maneuver. Further, it is discouraged and/or unreliable in postsurgical, abdominal hypertension, or fractured patients. We performed point-of-care echocardiography to address ejection fraction in all patients. However, we did not record the ratio of early transmitral flow velocity to the early diastolic velocity of the mitral annulus (E/E'); therefore, the incidence of elevated left ventricular filling pressures was unknown. This could be a possible bias in the study, as an elevated E/E’ (>15) is negatively correlated with lower performance at prediction of fluid responsiveness [35, 36]. In order to minimize time, we performed carotid measurements on a single respiratory cycle; therefore, we do not know if the accuracy could have been improved with the average of three respiratory cycles.

Although the reliability of PiCCO system has been found to be good in heterogeneous groups of patients, it is still questioned, mainly on the time-dependent accuracy on recalibrations and its variability on agreement at different vasopressor dose [30, 37, 38]. These data were not addressed in our study.

External validity is limited, as physicians involved at estimation of ΔCDPV were trained in critical care sonography for more than 1 year. Also, due to the observational nature of the study, management was not ΔCDPV-guided, and the spectrum of patients was narrow, including septic shock patients only. As long as there are clinical trials addressing these issues, our results should be interpreted cautiously. For statistical comparison between ROC curves with the Hanley–McNeil test, “measurements” are required. Therefore, we do not consider the relatively small sample size to be a limitation of our study.

## Conclusions

In this single-center study, we showed that ΔCDPV could be more accurate than other methods for assessing fluid responsiveness in patients with septic shock receiving lung protective mechanical ventilation. The ΔCDPV also has a high correlation with SVI increase after fluid challenge.

## Abbreviations

ΔCDPV: Carotid Doppler peak systolic velocity variation
AKI: Acute kidney injury
ARDS: Acute respiratory distress syndrome
AUROC: Area under the receiver operating characteristic curve
BSA: Body surface area
CAD: Coronary artery disease
CVP: Central venous pressure
GEDI: Global end-diastolic index
HR: Heart rate
ICU: Intensive care unit
IVC-d: Inferior vena cava diameter
ΔD-IVC: Respiratory variation in inferior vena cava diameter
LVOT: Left ventricle outflow tract
MAP: Mean arterial pressure
MaxCDPV: Maximum carotid peak velocity
MinCDPV: Minimum carotid peak velocity
NE: Norepinephrine
NPV: Negative predictive value
PEEP: Positive end-expiratory pressure
PiCCO: Pulse contour cardiac output system
PLR: Passive leg rising
PPV: Pulse pressure variation
PPV*: Positive predictive value
ROC: Receiver operating characteristic
RR: Relative risk
ScvO2: Oxygen saturation at central venous blood
SOFA: Sequential Organ Failure Assessment Score
SVI: Stroke volume index
SVV: Stroke volume variation
UO: Urinary output
VTI: Velocity time integral
